# Carbamoyl Phosphate Synthetase Subunit MoCpa2 Affects Development and Pathogenicity by Modulating Arginine Biosynthesis in *Magnaporthe oryzae*

**DOI:** 10.3389/fmicb.2016.02023

**Published:** 2016-12-19

**Authors:** Xinyu Liu, Yongchao Cai, Xi Zhang, Haifeng Zhang, Xiaobo Zheng, Zhengguang Zhang

**Affiliations:** Key Laboratory of Integrated Management of Crop Diseases and Pests, Ministry of Education, Department of Plant Pathology, College of Plant Protection, Nanjing Agricultural UniversityNanjing, China

**Keywords:** *Magnaporthe oryzae*, carbamoyl phosphate synthetase, arginine biosynthesis, conidiation, pathogenicity

## Abstract

Arginine is a semi-essential amino acid that affects physiological and biochemical functions. The *CPA2* gene in yeast encodes a large subunit of arginine-specific carbamoyl phosphate synthetase (CPS) and is involved in arginine biosynthesis. Here, an ortholog of yeast *CPA2* was identified in the rice blast fungus *Magnaporthe oryzae*, and was named *MoCPA2*. MoCpa2 is an 1180-amino acid protein which contains an ATP grasp domain and two CPSase domains. Targeted deletion of *MoCPA2* supported its role in *de novo* arginine biosynthesis in *M. oryzae* as mutant phenotypes were complemented by arginine but not ornithine. The Δ*Mocpa2* mutant exhibited defects in asexual development and pathogenicity but not appressorium formation. Further examination revealed that the invasive hyphae of the Δ*Mocpa2* mutant were restricted mainly to the primary infected cells. In addition, the Δ*Mocpa2* mutant was unable to induce a plant defense response and had the ability to scavenge ROS during pathogen-plant interactions. Structure analysis revealed that the ATP grasp domain and each CPS domain were indispensable for the proper localization and full function of MoCpa2. In summary, our results indicate that MoCpa2 plays an important role in arginine biosynthesis, and affects growth, conidiogenesis, and pathogenicity. These results suggest that research into metabolism and processes that mediate amino acid synthesis are valuable for understanding *M. oryzae* pathogenesis.

## Introduction

Phytopathogenic fungi cause diseases in humans, animals, and plants, and their ability to access the rich nutrient supply offered by living plants is one of the most obvious properties that distinguish pathogens from saprophytes (Divon and Fluhr, 2007). To survive in environments with limited and variable resources, pathogens have developed elegant and efficient genetic regulatory systems that enable them to respond rapidly to fluctuating nutritional conditions. Successful infection by pathogens depends as much on their ability to utilize the available nutrient sources offered by plants as on their ability to penetrate plants and evade defensive mechanisms (Solomon et al., 2003).

The hemi-biotrophic ascomycete *Mangnaporthe oryzae* has genetic regulatory systems that allow it to respond to nutrient quality and quantity in the environment and to complete the infection cycle. The regulatory systems include nitrogen metabolite repression (NMR) and carbon catabolite repression (CCR) to ensure the use of preferred sources of nitrogen (ammonium and L-glutamine) and carbon (glucose), respectively (Wilson et al., 2007, 2012; Wilson and Talbot, 2009; Fernandez et al., 2012). Both NMR and CCR are controlled by a Tor signaling pathway that regulates growth in response to nutrient availability (Franceschetti et al., 2011). Previous studies have shown that several genes involved in fungal metabolism are important to the infection-related morphogenesis and pathogenicity of *M. oryzae*. Cystathionine beta-lyase (MoStr3) and methylene tetrahydrofolate reductase (MoMet13) affect growth and development processes by regulating methionine biosynthesis (Wilson et al., 2012; Yan et al., 2013). SAICAR synthetase (MoAde1) is required for *de novo* adenine biosynthesis and pathogenicity (Fernandez et al., 2013). Orotate phosphoribosyl transferase (MoPyr5) is involved in uridine 5′-phosphate synthesis and controls the virulence of *M. oryzae* (Qi et al., 2016). An aminoadipate reductase (MoLys2) and a homocitrate synthase (MoLys20), regulated by G protein signaling regulators (Rgs), affect lysine biosynthesis and are important for the development and virulence of *M. oryzae* (Chen et al., 2014; Zhang et al., 2014). Acetolactate synthases (MoIlv2 and MoIlv6) are involved in leucine, isoleucine, and valine biosynthesis, and threonine deaminase (MoIlv1) is involved in isoleucine biosynthesis. All three of these enzymes control the growth, asexual development, and pathogenicity of the rice blast fungus (Du et al., 2013, 2014).

Arginine has a remarkable effect on physiological and biochemical function. In *Escherichia coli*, carbamoyl phosphate is the first precursor of the arginine biosynthetic pathway (Cunin et al., 1986). Carbamoyl phosphate is synthesized from glutamine, bicarbonate, and two molecules of ATP by carbamoyl phosphate synthetase (CPS; Hilger et al., 1973; Pierard and Schroter, 1978). In *S. cerevisiae*, arginine biosynthesis starts from glutamate and acetyl coenzyme A and first produces ornithine through the five acetylated steps with the acetyl group being recycled (Crabeel et al., 1997). Ornithine reacts with carbamoyl phosphate in the ornithine carbamoyl transferase step resulting in citrulline. Citrulline is then transformed to argininosuccinic acid, which is cleaved by lyase to form arginine (Slocum, 2005). *ScCPA2*, which encodes the large subunit of arginine-specific CPS, can use NH_3_ to synthesize bicarbonate and ATP (Pierard and Schroter, 1978; Crabeel et al., 1997). Carbamoyl phosphate is produced by a branch of the *de novo* arginine biosynthesis pathway, and is synthesized and utilized to directly supplement ornithine (Pierard and Schroter, 1978; Davis, 1986), although the importance of arginine biosynthesis in organisms is still unknown. Zhang et al. reported that three synthetic enzyme genes *MoARG1, MoARG5,6*, and *MoARG7* were involved in arginine biosynthesis and were required for the development and virulence of *M. oryzae* (Zhang et al., 2015). Therefore, further studies need to be conducted to explore the underlying mechanisms of arginine biosynthesis pathway in phytopathogens. Here, we identified and characterized the arginine-specific CPS subunit MoCpa2 in *M. oryzae*, and found that MoCpa2 appears to be involved in arginine biosynthesis and affects the development and virulence of the rice blast fungus.

## Materials and methods

### Fungal strains and culture conditions

The wild-type *M. oryzae* strain Guy11 and all generated strains were cultured on complete medium (CM) agar plates at 28°C (Talbot et al., 1993). Fungal mycelia were harvested from liquid CM and used for genomic DNA and RNA extractions. Protoplasts were prepared and transformed as described previously (Sweigard et al., 1992). Transformants were selected on TB3 medium with 250 mg/mL hygromycin B or 200 mg/mL bleomycin (Invitrogen, Carlsbad, CA, USA). For conidiation, mycelial blocks were maintained on straw decoction and corn agar medium (SDC) at 28°C for 7 days in the dark followed by 3 days of continuous illumination under fluorescent light (Zhang et al., 2009). For conidial production analysis, conidia were harvested and counted with a haemacytometer under a microscope. The final result of each sample = total number of conidia/colony area. Vegetative growth of Guy11, Δ*Mocpa2* mutant, and complemented strains was measured on CM, minimal medium (MM), and SDC medium for 7 days. Mycelial plugs were placed onto the freshly prepared MM agar plates with a range of concentrations of arginine or ornithine and cultured in the dark at 28°C for 7 days.

### Target gene deletion and complementation in *M. oryzae*

To generate a *MoCPA2* gene replacement construct, 1.0-kb upstream and 1.0-kb downstream flanking sequences were amplified from *M. oryzae* genomic DNA by PCR using the primer pairs FL04503F1 (F)/FL04503F2 (R) and FL04503F3 (F)/FL04503F4 (R), respectively. The upstream flanking sequences were digested by *Xho*I and *Eco*RI, and cloned into a pCX62 vector containing a hygromycin B cassette to generate the plasmid pCX62-*MoCPA2*-UP. The plasmid pCX62-*MoCPA2*-UP and the downstream flanking sequence were digested by *Spe*I and *Sac*I and ligated to generate the final construct pCX62-*MoCPA2*-U-D. Amino acid sequence alignments were performed using the CLUSTAL_W program. For complementation, the fragments containing the full-length coding region and a 1.5-kb native promoter region of the gene were amplified using primers FL04503F /FL04503R, and inserted into pYF11 containing bleomycin resistance and green fluorescent protein (GFP) genes to generate the pYF11-*MoCPA2-GFP* fusion construct, which was then transformed into protoplasts of the mutant. The complemented strains were screened by bleomycin resistance and GFP fluorescence observation. For construction of MoCPA2 domain deletion mutants, the fragments containing different domain deletion coding region and their 1.5-kb native promoter region were amplified using primers in Table S1, and inserted into pYF11 containing bleomycin resistance and GFP. The resulting constructs were then transformed into protoplasts of the mutant. The transformants were screened by bleomycin resistance and GFP fluorescence observation.

### Plant infection assays

For a rice spraying assay, conidial suspensions (5 × 10^4^ spores/mL) were sprayed onto 2-week-old rice seedlings and placed in a moist chamber at 28°C for 24 h in darkness. They were then transferred to another moist chamber with a 12-h light/12-h dark photoperiod under fluorescent lights. Disease severity was assessed by taking photographs of 6-cm rice blades 7 days after inoculation. For a detached barley infection assay, conidial suspensions (10^5^, 10^4^, and 10^3^ spores/mL) with or without arginine were dropped onto the barley leaves, and photographed after 5 days. For a rice sheath assay, 100 μL of conidial suspensions (5 × 10^4^ spores/mL) were inoculated into the inner leaf sheath cuticle cells. After 48 h of incubation under humid conditions at 28°C, the leaf sheaths were observed under a microscope (Zhang et al., 2011).

### DAB staining assay

To observe the plant defense response, infected leaf-sheath cells were prepared as described above (Qi et al., 2015) and examined under a microscope with ultraviolet radiation. Host derived reactive oxygen species (ROS) staining was performed as previously described (Chi et al., 2009). The experiments were performed in three independent biological experiments with three replicates.

### Nucleic acid manipulation, qRT-PCR, and southern blot assays

The target gene probe and *HPH* probe were amplified with the primer pairs FL04503cF/FL04503cR (for *MoCPA2*) and FL1111/FL1112 (for *HPH*), respectively. Probe labeling, hybridization, and detection were performed with a DIG High Prime DNA Labeling and Detection Starter Kit (Roche Applied Science, Penzberg, Germany). Total RNA was isolated from frozen fungal mycelia using an RNA extraction kit (Invitrogen, USA). To measure the relative abundance of gene transcripts, RNAs were extracted from mycelia grown in CM liquid medium for 2 days at 28°C in a 150-rpm orbital shaker (Guo et al., 2011). To measure the relative abundance of *MoCPA2* transcripts during diverse fungal development stages, the total RNA samples were extracted from mycelia grown in CM liquid medium, from conidia, and from plants inoculated with the Guy11 conidia (1 × 10^8^ spores/mL) for 8, 24, 48, and 72 h by the method described above (Guo et al., 2010; Dong et al., 2015). Total RNA was pretreated with DNase I and was then reverse transcribed (Invitrogen, Carlsbad, CA, USA). For the evaluation of transcription level of PR genes, RNA samples were extracted from the rice leaves inoculated with Guy11, the mutant (1 × 10^8^ spores/mL) and water (mock) for 48 h. qRT-PCR was performed on an ABI 7500 real-time PCR system (Applied Biosystems, Foster City, CA, USA) according to the manufacturer's instructions. The stable-expression *ACTIN* gene (MGG_03982) amplified by primers FL04503RTF and FL04503RTR was used as internal control. All primers used in this study are listed in Table S1.

### Statistical analysis

Each result was presented as the mean ± standard deviation (SD) of at least three replicated measurements. The significant differences between treatments were statistically evaluated by SD and one-way analysis of variance (ANOVA) using SPSS 2.0. The data between two specific different treatments were compared statistically by ANOVA, followed by *F*-test if the ANOVA result is significant at *P* < 0.05 and *P* < 0.01.

## Results

### Identification and expression of MoCpa2 in *M. oryzae*

**Figure 1:**
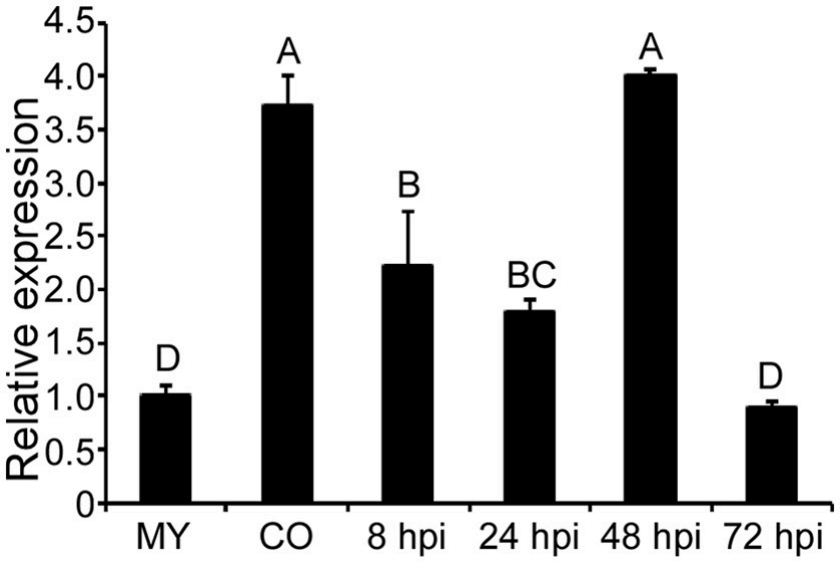
**Transcription profiles of ***MoCPA2*** at different development stages of ***M. oryzae*****. The stage-specific expression of *MoCPA2* was quantified by qRT-PCR. Total RNA was extracted from mycelia (MY), conidia (CO), and infectious hyphae of the wild type Guy11. Three independent experiments yielded similar results. Error bars represent standard deviation. Uppercase Letters represents the significant differences between the different samples.

*MoCPA2* (MGG_04503) was identified in the *M. oryzae* genome database as a homolog of yeast *CPA2* which encodes a CPS. (http://www.broadinstitute.org/annotation/genome/magnaporthe_comparative/MultiHome.html). To investigate the role of *MoCPA2* in *M. oryzae*, we first examined its transcript levels at various pathogen developmental stages using qRT-PCR analysis. Compared to the mycelial stage, the *MoCPA2* transcript level was much higher in the conidial and early infectious stages, with a 2.7-fold increase in the conidial stage and a one- to three-fold increase in the early infectious stages at 8, 24, and 48 h post-infection (hpi). However, there were no expression differences in late infectious stages (72 hpi; Figure 1). These results suggested that *MoCPA2* likely plays an important role in asexual development and early infection in the rice blast fungus. To further examine the biological function of *MoCPA2*, the *MoCPA2* coding region was replaced with the hygromycin resistance cassette (*HPH*) via homologous recombination, and the successful gene deletion mutant was confirmed by Southern blot analysis (Figure S1).

### MoCpa2 is involved in arginine biosynthesis

**Figure 2:**
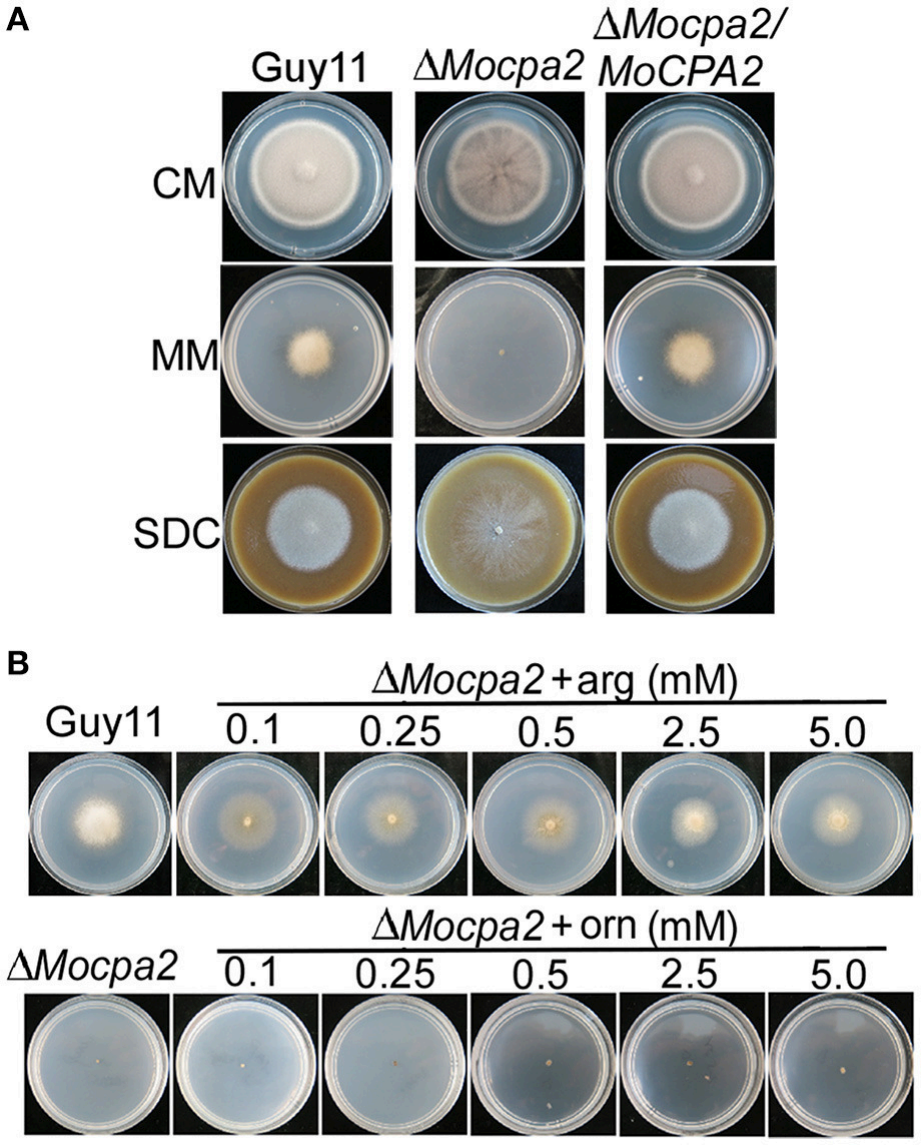
**Defect of the Δ***Mocpa2*** mutant in mycelial growth and exogenous arginine but not ornithine could restore the defect**. **(A)** Colony morphology of the wild type Guy11, Δ*Mocpa2* mutant and complemented transformant on CM, MM, and SDC media. **(B)** The Δ*Mocpa2* mutant was inoculated on MM with/without different concentrations of arginine (arg) or ornithine (orn) and cultured at 28°C for 7 days.

To clarify the role of MoCpa2 in *M. oryzae*, we first observed the vegetative growth of the Δ*Mocpa2* mutant on CM, MM, and SDC media. After 7 days incubation, no significantly difference in colony diameter was observed between Guy11 and the Δ*Mocpa2* mutant on CM and SDC plates, but the mutant showed fewer aerial hyphae under the same conditions (Figure 2A). In contrast, the Δ*Mocpa2* mutant could not grow on MM plates (Figure 2A). In yeast, Cpa2 is known to catalyze the synthesis of an arginine precursor and is involved in arginine biosynthesis (Pierard and Schroter, 1978). Therefore, MM plates with various concentrations of arginine were prepared to test the growth of the mutant. The results showed that exogenous arginine was able to restore the growth rate but not the aerial hyphae of the mutant at low concentrations (Figure 2B). The Δ*Mocpa2* mutant displayed more aerial hyphae and became grayish white when the arginine concentration was increased, which was similar to that of the wild-type Guy11 strain at concentrations of 2.5 and 5.0 mM (Figure 2B). As the ornithine reacts with carbamoyl phosphate in the ornithine carbamoyl transferase step (Hilger et al., 1973; Pierard and Schroter, 1978), and is the precursor of arginine, we also added different concentrations of ornithine into MM medium to test the growth of the mutant. The results showed that exogenous ornithine was unable to restore the growth defects of the Δ*Mocpa2* mutant (Figure 2B). These results indicated that MoCpa2 plays a role in arginine utilization. Since nitric oxide synthase was known to be involved in the utilization of arginine (Durante et al., 2007). We further analyzed the activity of nitric oxide synthase in the mutant. The results revealed that the activity of nitric oxide synthase was decreased to 48.2 and 27.5% in the Δ*Mocpa2* mutant at 24 and 48 h post incubation, respectively (Figure S2), indicating that MoCpa2 is involved in the utilization of arginine medicated by the activity of nitric oxide synthase in *M. oryzae*.

### MoCpa2 plays an essential role in asexual development

**Figure 3:**
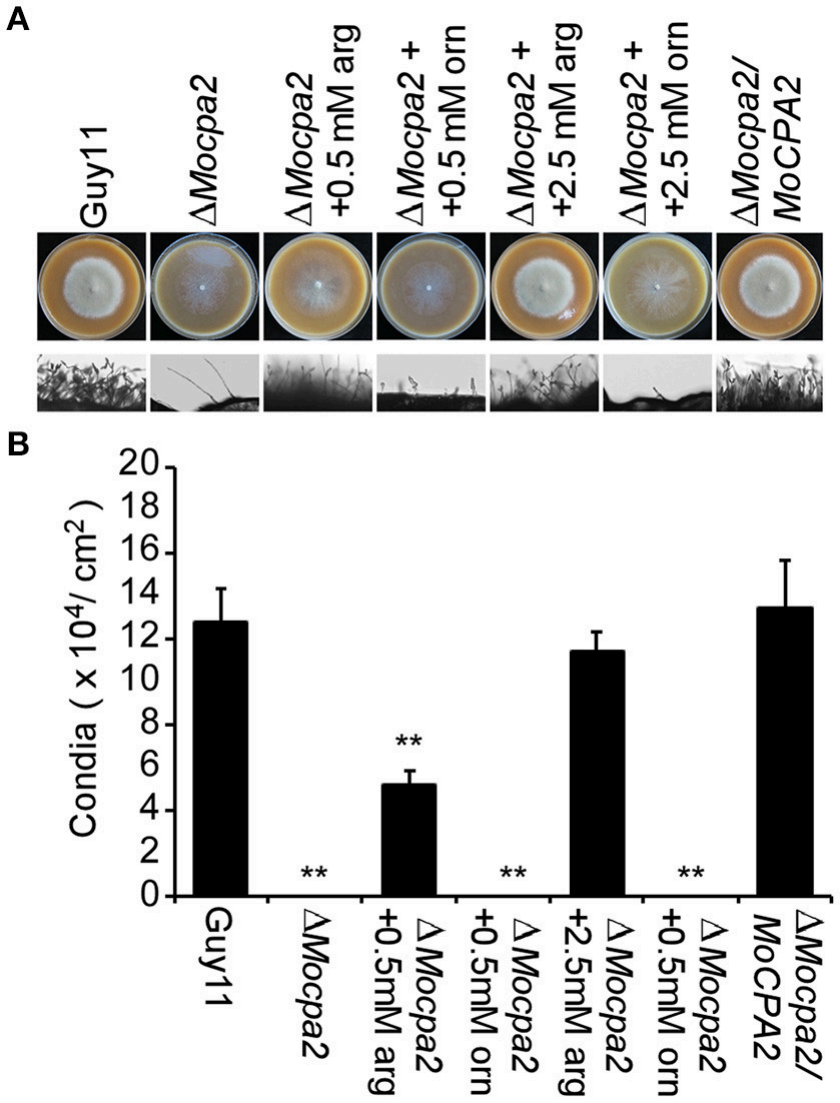
**The Δ***Mocpa2*** mutant was defective in conidiation**. **(A)** Colony morphology and conidial development on SDC media with or without arginine or ornithine. Photographs for plates were taken at 7 days post-inoculation (dpi). **(B)** Statistical analysis of conidial production. Error bars represent the standard deviations and asterisks denote statistical significances (*P* < 0.01).

Conidia are important structures for *M. oryzae* to infect the host plant (Kim et al., 2009; Liu et al., 2010). Therefore, we evaluated the ability of the Δ*Mocpa2* mutant to produce conidia by incubation the Δ*Mocpa2* mutant on SDC plates for 7 days. No conidia were collected from the Δ*Mocpa2* mutant compared with a large number of conidia produced by the wild-type Guy11 and complemented strains under the same conditions (Figures 3A,B), indicating that the Δ*Mocpa2* mutant was defective in asexual development. To determine whether this defect was related to arginine, exogenous arginine was added to SDC plates. In the presence of exogenous arginine, conidia production in the mutant was restored to 53 and 93% of that of the wild-type strains with arginine concentrations of 0.5 and 2.5 mM, respectively (Figures 3A,B). Meanwhile, exogenous ornithine was also added to the SDC plates, and no conidia were harvested even the concentration of ornithine increased to 2.5 mM (Figures 3A,B). These results indicate that MoCpa2 plays an essential role in conidiogenesis by controlling arginine biosynthesis in *M. oryzae*.

### MoCpa2 is important for pathogenicity but not appressorium formation

**Figure 4:**
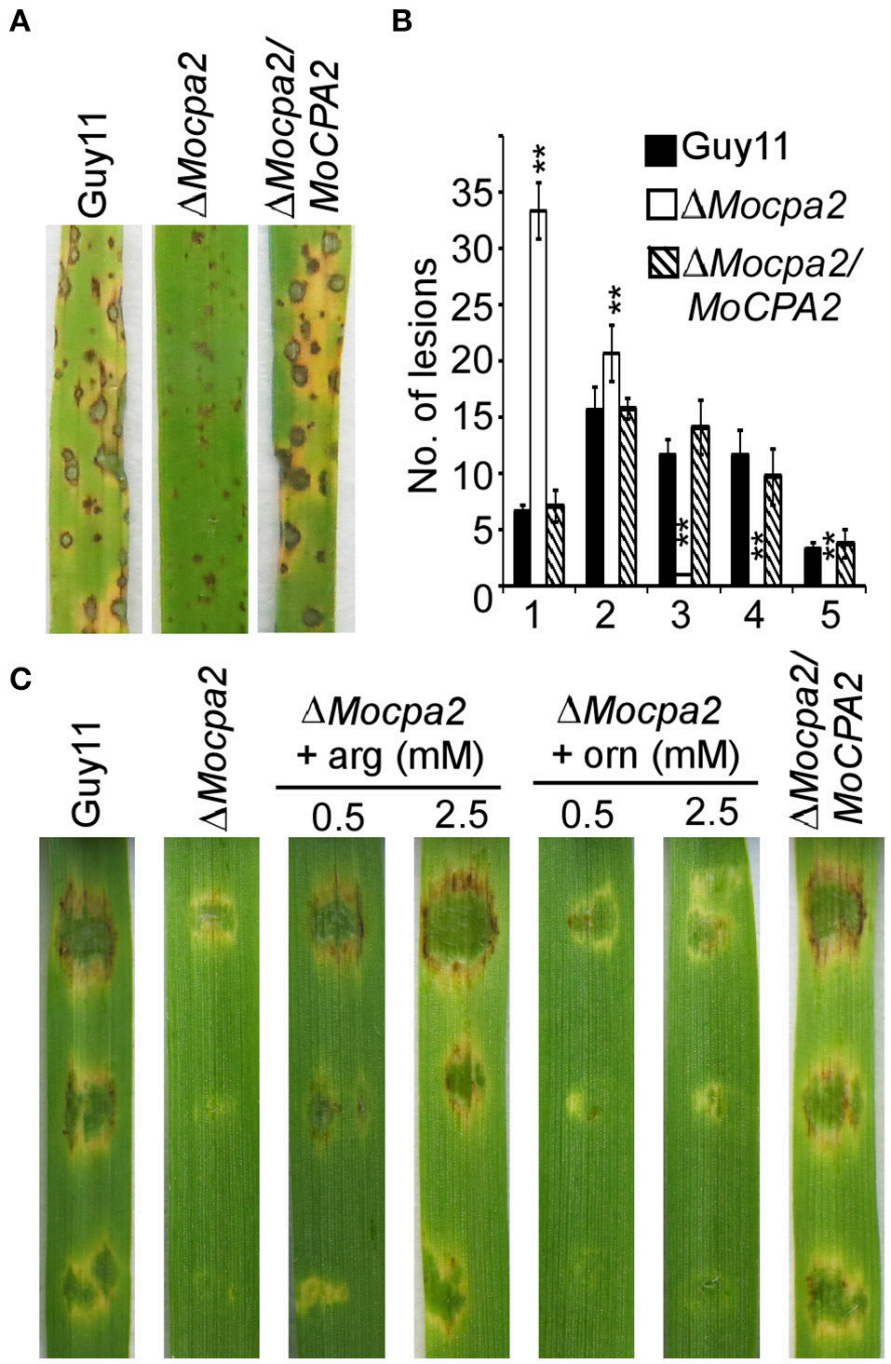
**Deletion of ***MoCPA2*** attenuates pathogenicity. (A)** Conidia suspensions from the wild type Guy11, Δ*Mocpa2* mutant and complemented transformant were sprayed onto the rice seedlings. Photographs were taken at 7 dpi. **(B)** Quantification of lesion types caused by the indicated strains (0, no lesion; 1, pinhead-sized brown specks; 2, 1.5-mm brown spots; 3, 2–3-mm gray spots with brown margins; 4, many elliptical gray spots longer than 3 mm; 5, coalesced lesions infecting 50% or more of the leaf area). Lesions were measured within an area of 4 cm^2^. Three independent biological experiments yielded similar results. Error bars represent standard deviation, and asterisks represent significant difference between Guy11 and the mutant (*P* < 0.01). **(C)** Conidial suspensions (with/without arginine or ornithine) from the indicated strains were dropped onto the detached barley leaves. Photographs were taken at 5 dpi.

To gain insight into the functions of MoCpa2 in pathogenicity, Δ*Mocpa2* mutant and wild-type conidia were collected from SDC plates containing 2.5 mM arginine and sprayed onto 2-week-old rice seedlings. The Δ*Mocpa2* mutant caused tiny and restricted lesions in comparison to the typical blast lesions caused by the wild-type Guy11 and complemented strains (Figure 4A). A “lesion-type” scoring assay also confirmed this result. The mutant showed more type 1 and type 2 lesions, and caused fewer or no type 3–5 lesions (Figure 4B). These results suggested that even the conidia recovered by arginine cannot infect the host normally. Therefore, arginine as well as ornithine was added to conidial suspensions and inoculated onto detached barley leaves. The results showed that exogenous ornithine was unable to restore the virulence defects of the Δ*Mocpa2* mutant. In contrast, 0.5 mM arginine could partially restore the virulence of the mutant, while 2.5 mM arginine could completely restore this defect on barley leaves (Figure 4C). However, the defect was not suppressed on rice seedlings through the spraying assay (Figure S3A). These results suggest that MoCpa2 is important for pathogenicity by controlling arginine biosynthesis in the rice blast fungus. We also examined appressorium formation on an inductive surface, and found appressorium formation was not altered in the Δ*Mocpa2* mutant (Figure S3B).

### MoCpa2 is involved in infectious hyphal growth

**Figure 5:**
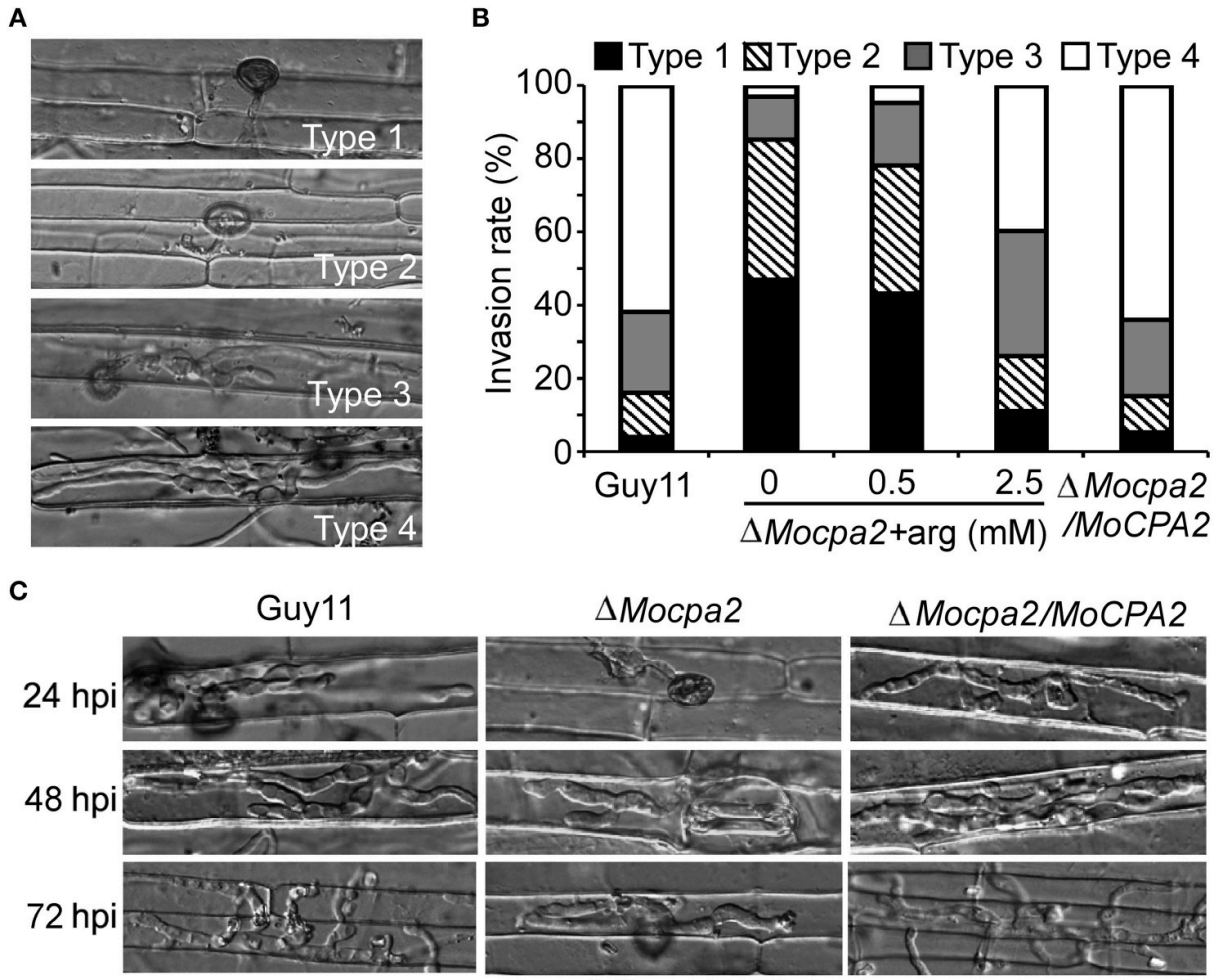
**Defects of the Δ***Mocpa2*** mutant in infectious hyphae growth. (A,B)** Statistical analysis of the infectious hyphal type (type 1, no penetration; type 2, with penetration peg; type 3, with a single invasive hypha; type 4, with extensive hyphal growth) on rice leaf sheaths. Rice leaf sheaths were inoculated with conidial suspensions with or without arginine and examined at 36 h post-inoculation (hpi). Hundred infected hyphae were counted for each strain and the experiment was repeated three times. **(C)** Rice leaf sheath infection assay. The infectious hyphal growth was observed at 24, 48, and 72 hpi, respectively.

To determine why the Δ*Mocpa2* mutant attenuates pathogenicity, we assayed infectious hyphae growth in rice sheath leaves. Four types of invasive hyphae were observed and analyzed. In the wild-type Guy11 and the complemented strains, over 80% of cells showed type 4 lesions, and fewer than 20% showed type 1 and type 2 lesions. In contrast, 15% of cells showed type 3 and type 4 lesions, and 85% showed type 1 and type 2 lesions in the Δ*Mocpa2* mutant (Figures 5A,B), indicating that the Δ*Mocpa2* mutant was defective in infectious hyphae growth. We further added exogenous arginine to conidial suspensions of the mutant, and found that the defect could be greatly restored by 2.5 mM arginine, but not by 0.5 mM arginine (Figures 5A,B). Infectious growth on rice sheathes was also observed by time course. The results showed that the Δ*Mocpa2* mutant rarely penetrated through the rice cuticle at 24 hpi, and that the invasive hyphae remained restricted to the primary infected cells at 48 and 72 hpi. In contrast, the invasive hyphae of the wild-type and complemented stains extended into neighboring cells under the same conditions (Figure 5C). These results suggest that MoCpa2 plays a critical role in penetration and infectious hyphal growth by affecting arginine biosynthesis, thus affecting pathogenicity.

### Impaired arginine biosynthetic pathway results in the development and virulence defects of *M. oryzae*

**Figure 6:**
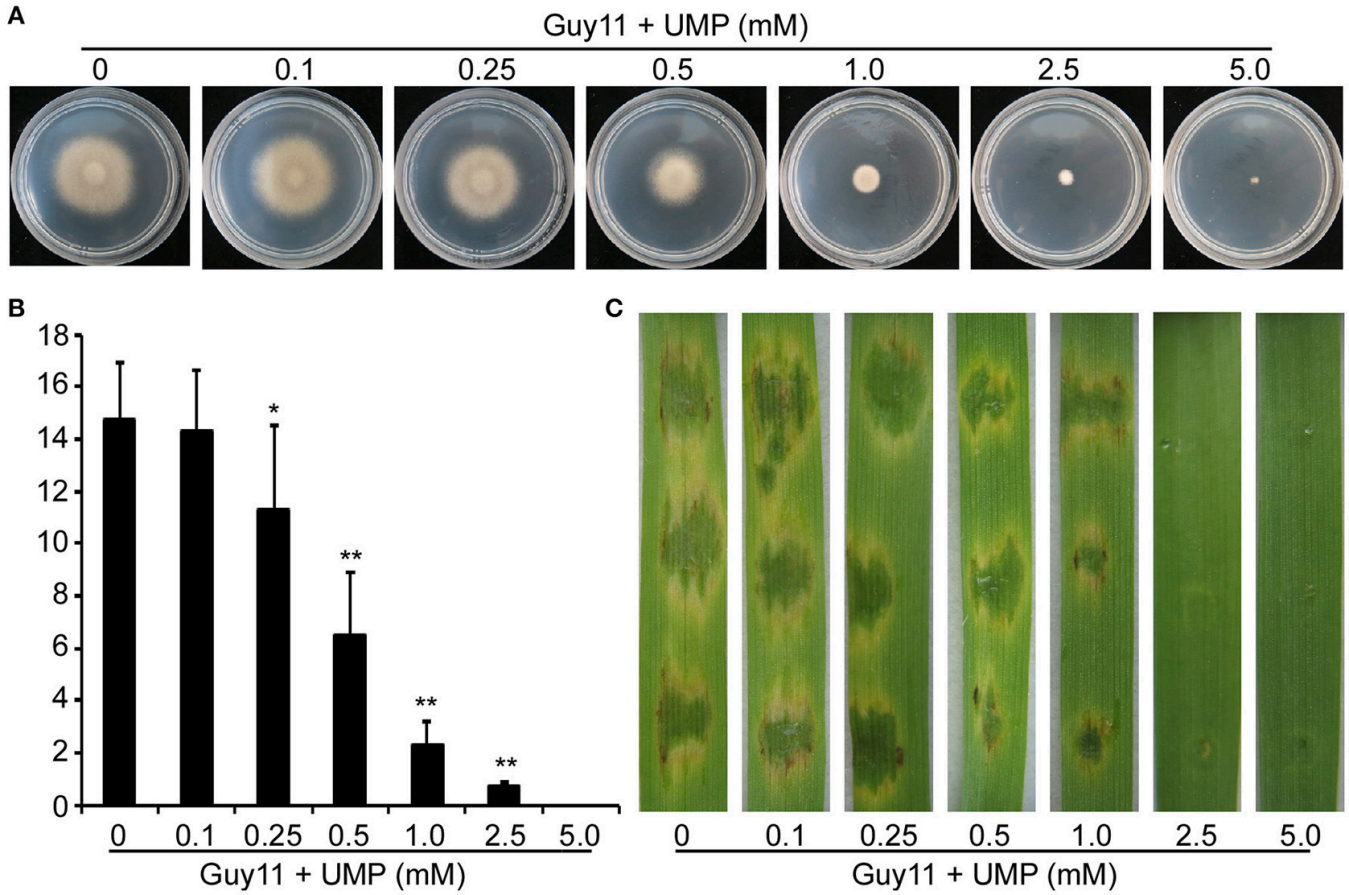
**UMP inhibits the vegetative growth, conidiation, and pathogenicity of the wild type Guy11. (A)** The Guy11 was inoculated on MM with different concentrations of UMP and cultured at 28°C for 7 days. **(B)** Statistical analysis of conidial production. The conidia produced by Guy11 grown on SDC medium with UMP (0–5 mM) for 10 days were collected and analyzed. Three independent biological assays were performed and similar results were obtained. The error bars present standard deviation and the asterisks indicate a significant difference among different treatments (^*^P < 0.05, ^**^P < 0.01). **(C)** Conidial suspensions (with or without UMP) from the Guy11 were dropped onto the detached barley leaves. Photographs were taken at 5 dpi.

The large subunit of CPS is responsible for binding to the inhibitor uridine monophosphate (UMP; Bueso et al., 1999). To further test the observed defects of the Δ*Mocpa2* mutant are due to arginine limitation or as a result total pathway disruption, the wild type Guy11 was treated with different concentrations of UMP. Growth rate of Guy11 was decreased under the treatment of UMP, and completely inhibited on MM plates with 5 mM UMP (Figure 6A). We also examined conidiation and found that on SDC medium with different concentrations of UMP, conidial production of Guy11 was significantly reduced, and was unable to produce conidia under 5 mM UMP (Figure 6B). Further infection assay on barley leaves showed that 0.5 mM UMP could cause defects on virulence of Guy11, when the concentration of UMP increased to 5 mM, the infection ability of Guy11 was completely abolished on barely leaves (Figure 6C). These results indicated that arginine biosynthetic pathway mediated by MoCpa2 is important for vegetative growth, asexual development, and pathogenicity of the rice blast fungus.

### Deletion of *MoCpa2* does not elicit plant defense responses

**Figure 7:**
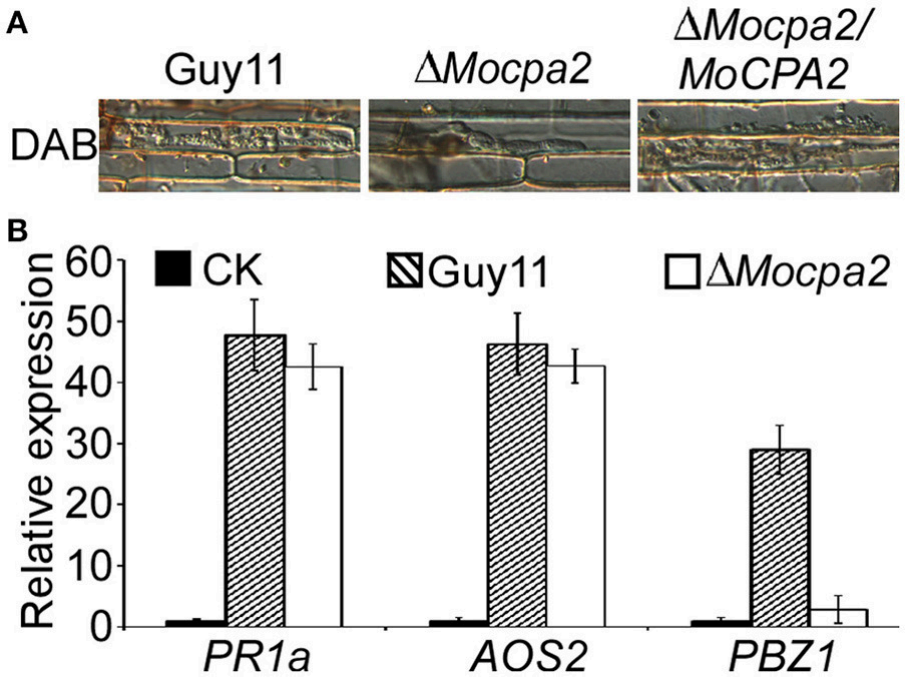
**The Δ***Mocpa2*** mutant was unable to induce plant defense response. (A)** DAB (3, 3′-Diaminobenzidine) staining of the rice sheath infected by Guy11, Δ*Mocpa2* mutant and the complement transformant at 48 hpi. The experiment was performed with three independent experiments. **(B)** qRT-PCR analysis of the transcription of *AOS2, PR1a*, and *PBZ1* in the infected rice leaves. RNA samples were collected from rice leaves inoculated with the wild type Guy11, Δ*Mocpa2* mutant (1 × 10^8^ spores/mL) and water (mock) for 48 h. The average threshold cycle of triplicate reactions was normalized by the stable-expressions gene *EF1a* (Os03g08020) in *O. sativa*. Three independent biological experiments were performed and yielded similar results. Error bars represent the standard deviation.

Plant defense responses are important during plant-microbe interaction. The recognition of microbe-associated molecules by plant immune systems results in the rapid generation of ROS in the infected cell, accumulation of pathogenesis-related (PR) proteins, and reinforcement of the cell wall (Bradley et al., 1992; Levine et al., 1994; Abramovitch et al., 2003; Tanaka et al., 2003; Torres and Dangl, 2005). Therefore, we speculated that the defects in infectious growth of the mutant partly resulted from the plant defense responses. ROS accumulation was examined by staining with 3,3′-diaminobenzidine (DAB) after inoculation for 48 h. Similar to the results in the wild-type Guy11 and complemented strains, no ROS accumulation was observed at the infection sites of the Δ*Mocpa2* mutant (Figure 7A). We further evaluated the expression of the PR genes, *PR1a, AOS2*, and *PBZ1*, which were previously shown by qRT-PCR analysis to be expressed at their highest levels during plant-pathogen interactions (Chi et al., 2009). The expression levels of the three PR genes did not increase in the Δ*Mocpa2* mutant compared to the wild-type Guy11 strain (Figure 7B), suggesting that deletion of *MoCPA2* did not induce their expression. We also found that the expression of *PBZ1* was much lower in rice inoculated with the Δ*Mocpa2* mutant compared to the wild type Guy11. As the expression of *PBZ1* in the Δ*Mocpa2* mutant showed no significant difference from the mock, indicating that deletion of *MoCPA2* did not induce the *PBZ1* expression by 48 hpi. Therefore, we concluded that the Δ*Mocpa2* mutant cannot elicit plant defense responses at early stages of infection, and that the attenuated virulence of the mutant does not due to plant defense responses.

### Functional characterization of MoCpa2 domains

**Figure 8:**
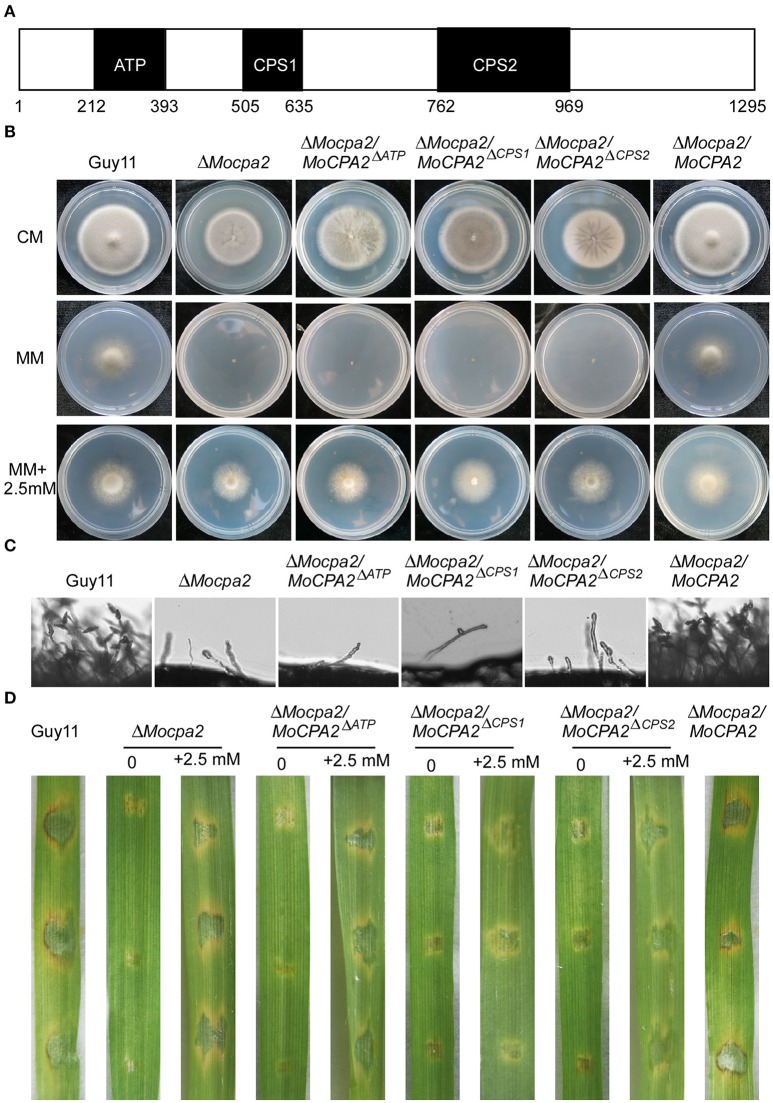
**Functional analysis of different domains of MoCpa2. (A)** Structure diagram of the MoCpa2. **(B)** Colony morphology and growth rate of the indicated strains on CM, MM with or without exogenous arginine. **(C)** Observation conidiophore and conidia induced on slide glasses. **(D)** Infection assay. Conidia suspensions (with or without 2.5 mM arginine) from the indicated strains were dropped onto the detached barley leaves. Photographs were taken at 5 dpi.

Structural analysis revealed that MoCpa2 contains an ATP grasp domain and two CPSase domains (Figure 8A). To investigate the roles of these three domains in *M. oryzae*, domain deletion constructs were fused with GFP to produce MoCpa2^ΔATP^-GFP (deletion of the ATP domain), MoCpa2^ΔCPS1^-GFP (deletion of the CPS1 domain), and MoCpa2^ΔCPS2^-GFP (deletion of the CPS2 domain) constructs which were transformed into the Δ*Mocpa2* mutant. The resulting transformants were evaluated for GFP signals and analyzed. The tranformants expressing MoCpa2^ΔATP^-GFP, MoCpa2^ΔCPS1^-GFP, and MoCpa2^ΔCPS2^-GFP showed growth, conidiation, and virulence phenotypes similar to those of the Δ*Mocpa2* mutant (Figures 8B–D). Further studies revealed that 2.5 mM exogenous arginine could restore the defects of each transformant (Figures 8B–D), suggesting that each domain is essential for the full function of MoCpa2 in *M. oryzae*. We also observed the localization of MoCpa2 and the domain deletion transformants. The results showed that MoCpa2 localized to mitochondria in *M. orzyae* (Figure S4A), and GFP signals of the domain deletion transformants were mainly observed in cytoplasm (Figure S4B), indicating ATP grasp domain and two CPSase domains are indispensable for proper localization of MoCpa2, thereby affecting the growth and development of the rice blast fungus.

## Discussion

Nutrient metabolism plays important roles in the growth and differentiation of *M. oryzae*, which blocks amino acid biosynthesis and causes defects in growth, asexuality, and virulence (Wilson et al., 2012; Du et al., 2013; Yan et al., 2013). In this study, we characterized the CPS large subunit, MoCpa2, in *M. oryzae* and found that the Δ*Mocpa2* mutant could not grow on MM plates. Moreover, this defect was suppressed by adding exogenous arginine but not ornithine, consistent with a role for MoCpa2 in *de novo* arginine biosynthesis. The targeted deletion of *MoCPA2* also caused defects in asexual development, infection-related morphogenesis, and virulence, all of which were restored by continuous exogenous arginine, suggesting that arginine plays an important role in regulating these processes. Simultaneously, the Δ*Mocpa2* mutant exhibited small lesions and a deficiency of typical infection spots, suggesting either that appressorium formation or initial infection ability is not affected by arginine, or that arginine is necessary in the appressorium stage and is finally depleted after appressorium formation and plant infection. By inhibiting the CPS in the wild type Guy11 with UMP, we further confirmed that the defects of Δ*Mocpa2* mutant were due to the total arginine biosynthesis pathway disruption, which plays critical roles in the development and virulence of the rice blast fungus. In addition, the activity of the nitric oxide synthase, which is involved in the utilization of arginine, was significantly decreased in the mutant; implicating CPS also plays a critical role in the utilization of arginine in *M. oryzae*.

The host defense response is an important mechanism by which the plant resists pathogen invasion, and includes the production of ROS. ROS scavenging by pathogens is one way organisms bypass plant responses to establish infections (Nathues et al., 2004; Molina and Kahmann, 2007; Chi et al., 2009). ROS are involved in host defense against *M. oryzae*, and the ability to eliminate intracellular ROS contributes to virulence (Molina and Kahmann, 2007). DAB was used to evaluate the greater amounts of H_2_O_2_ accumulated in rice cells around the appressorium and initially infected cells in *M. oryzae* (Guo et al., 2010; Liu et al., 2016). In addition, *PR* genes such as *PBZ1, PR1a*, and *AOS2*, are key components that involved in JA- and SA-induced plant defense (Mei et al., 2006; Qiu et al., 2007). The expressions of *PR* genes were also used to analyze the defense response of the host to the rice blast fungus (Guo et al., 2010, 2011; Liu et al., 2016). Our results showed that the interestinglyinterestinglyΔ*Mocpa2* mutant failed to elicit *PR* gene expression and scavenge the ROS induced by host. Based on these results, we hypothesized that the host defense response was not the major reason for the restricted growth of invasion by *M. oryzae* hyphae. A previous study showed that the SAICAR synthetase-encoding gene *MoADE1* is involved in the pathogenicity of *M. oryzae* but did not elicit plant defense response (Fernandez et al., 2013). Thus we focused on the synthesis of arginine and found that continuous exogenous arginine could suppress the pathogenicity defects of the interestingly Δ*Mocpa2* mutant on barley leaves, which was consistent with the excised leaf-sheath assay results. These results suggest that a lack of arginine caused the infection defect of *M. oryzae*. However, exogenous arginine was unable to suppress the virulence defects on rice seedlings in the spraying assay, suggesting that continuous arginine is essential for the infection by *M. oryzae*.

MoCpa2 domain characterization showed that each domain plays an essential role in maintaining the normal function of MoCpa2 during arginine biosynthesis, as well as conidiation and pathogenicity. Further localization observation revealed that MoCpa2 functions as a mitochondria protein and the domain deletion mutant changed the proper localization of MoCpa2 from mitochondria to cytoplasm, which resulted in similar phenotypes to the mutant. These findings suggested that each domain of MoCpa2 is indispensable for proper localization of MoCpa2, which is required for normal development and pathogenicity of *M. oryzae*. Taken together, our results support a crucial role for MoCpa2 in arginine biosynthesis, which is important for mycelial growth, asexual development, and pathogenicity of *M. oryzae*. These findings provide evidence for the importance of amino acid metabolism in the development of filamentous phytopathogens.

## Conclusion

This study characterized a carbomyl phosphate synthetase MoCpa2 and its impact on virulence. The findings support a role for MoCpa2 in arginine biosynthesis and show that this gene is important to infection morphogenesis development in the rice blast fungus. The findings revealed new insights into the underlying mechanisms of arginine biosynthesis pathway that may help to the identification of new targets for fungicide or the designment of new disease management strategies.

## Author contributions

Conceived and designed the experiments: XL, HZ, ZZ. Performed the experiments: XL, YC, XZhang. Analyzed the experiment data: XL, YC, XZhang, HZ. Contributed reagents/materials/analysis tools: XL, YC, XZhang, XZheng. Wrote the paper: XL, HZ, ZZ. All authors have read and approve the final manuscript.

### Conflict of interest statement

The authors declare that the research was conducted in the absence of any commercial or financial relationships that could be construed as a potential conflict of interest.

## Supplementary material

Figure S1 **Targeted deletion of ***MoCPA2*** in ***M. oryzae*****. Strategy of deletion *MoCPA2* gene in *M. oryzae* genome and Southern blot analysis of the gene knockout mutant.

Figure S2 **Analysis of the nitric oxide synthase activity of the Δ***Mocpa2*** mutant**. The activities of the nitric oxide synthase were evaluated by the Nitric Oxide Synthase Assay Kit (beyotime, China). Mycelia were cultured in liquid CM medium for 24 h or 48 h, and analyzed. Asterisks denote statistical significances (*P* < 0.01).

Figure S3 **Spraying assay on rice seedlings and appressorium formation assay. (A)** Conidial suspensions with different concentrations of arginine were sprayed onto the rice seedlings. Diseased leaves were photographed at 7 dpi. **(B)** Appressorium formation was allowed on inductive surfaces and observed at 24 hpi. The experiment was repeated three times for each strain to determine the mean value.

Figure S4 **Localization of MoCpa2 and its domain deletion mutants. (A)** Hyphae expressing the MoCpa2-GFP and stained by MitoTracker Red CMXRos were observed under a fluorescence microscope. **(B)** Hyphae expressing the domain deletion constructs were observed under a fluorescence microscope.

Table S1 **Primers used in this study**.
