# Supplementary figures and images for: Carbamoyl Phosphate Synthetase Subunit MoCpa2 Affects Development and Pathogenicity by Modulating Arginine Biosynthesis in Magnaporthe oryzae
Source: Front Microbiol. 2016 Dec 19;7:2023. doi: 10.3389/fmicb.2016.02023 (PMC5166579; doi:10.3389/fmicb.2016.02023)

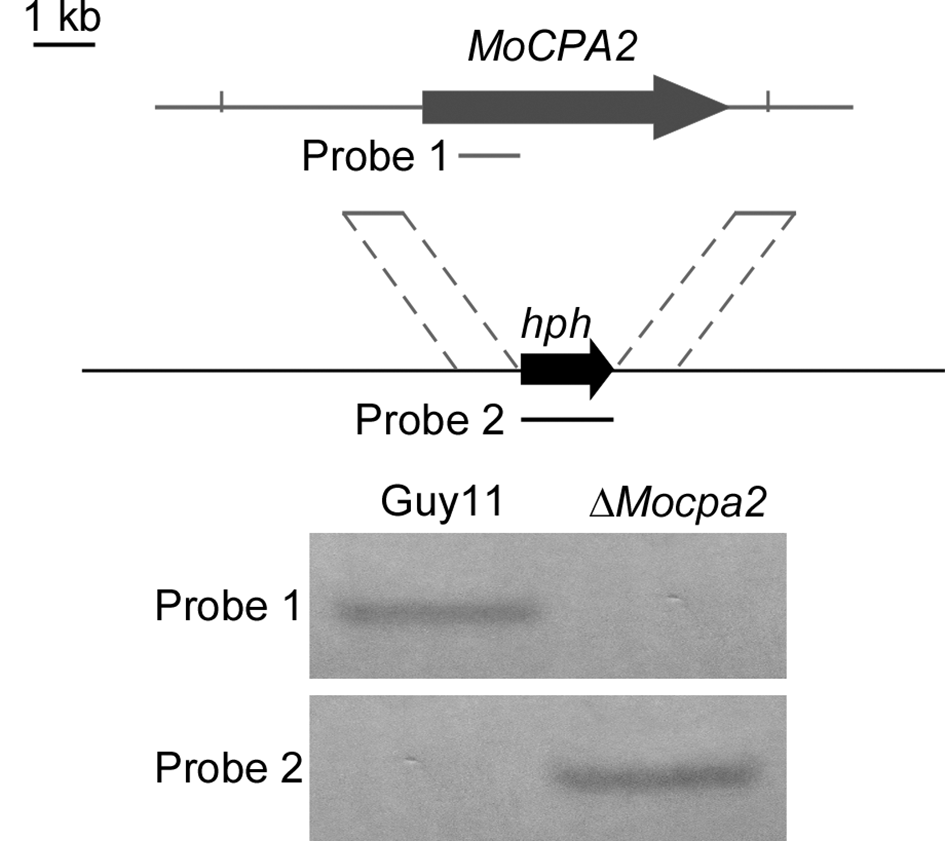

Supplement: Figure S1 — Targeted deletion of MoCPA2 in M. oryzae. Strategy of deletion MoCPA2 gene in M. oryzae genome and Southern blot analysis of the gene knockout mutant. [file Image1.TIF]

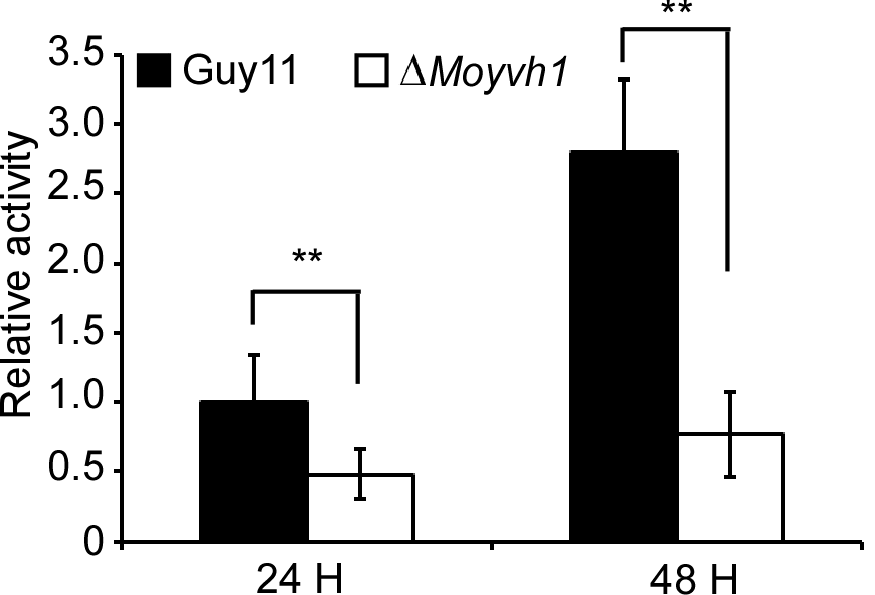

Supplement: Figure S2 — Analysis of the nitric oxide synthase activity of the ΔMocpa2 mutant. The activities of the nitric oxide synthase were evaluated by the Nitric Oxide Synthase Assay Kit (beyotime, China). Mycelia were cultured in liquid CM medium for 24 h or 48 h, and analyzed. Asterisks denote statistical significances (P < 0.01). [file Image2.TIF]

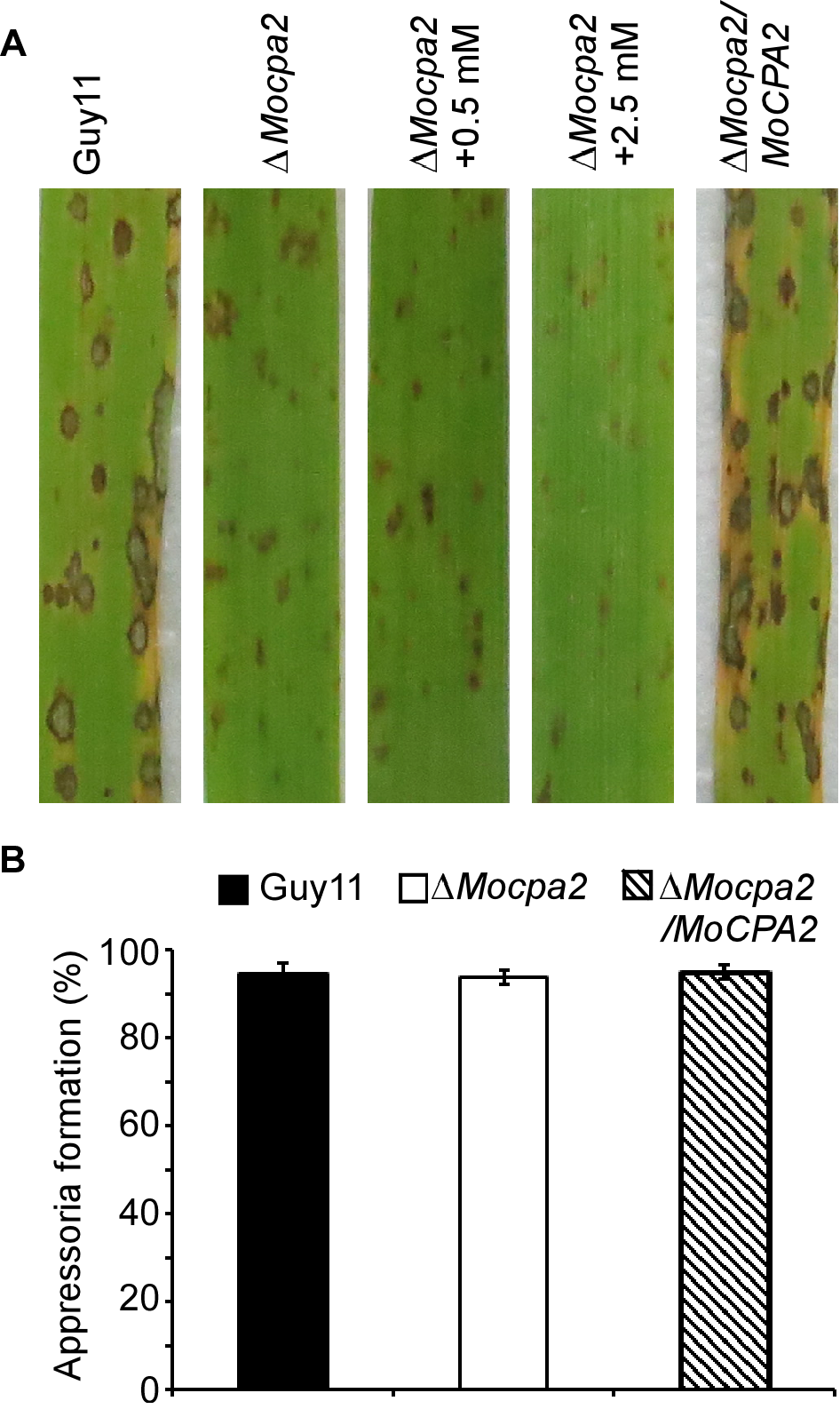

Supplement: Figure S3 — Spraying assay on rice seedlings and appressorium formation assay. (A) Conidial suspensions with different concentrations of arginine were sprayed onto the rice seedlings. Diseased leaves were photographed at 7 dpi. (B) Appressorium formation was allowed on inductive surfaces and observed at 24 hpi. The experiment was repeated three times for each strain to determine the mean value. [file Image3.TIF]

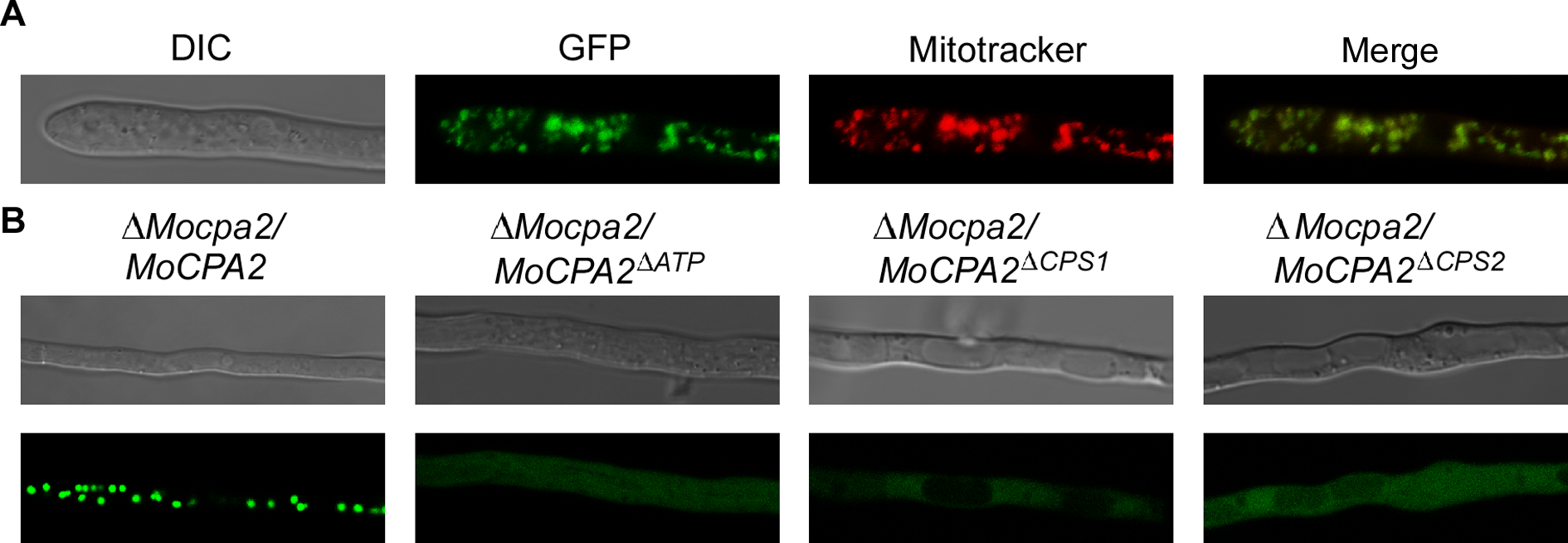

Supplement: Figure S4 — Localization of MoCpa2 and its domain deletion mutants. (A) Hyphae expressing the MoCpa2-GFP and stained by MitoTracker Red CMXRos were observed under a fluorescence microscope. (B) Hyphae expressing the domain deletion constructs were observed under a fluorescence microscope. [file Image4.TIF]
