# Supplementary material for: Carbamoyl Phosphate Synthetase Subunit MoCpa2 Affects Development and Pathogenicity by Modulating Arginine Biosynthesis in Magnaporthe oryzae
Source: Front Microbiol. 2016 Dec 19;7:2023. doi: 10.3389/fmicb.2016.02023 (PMC5166579; doi:10.3389/fmicb.2016.02023)
Supplement: Table S1 — Primers used in this study. [file Table1.DOCX]

**Table S1. Primers used in this study**

| **Primer name** | **Sequence (5’-3’)** | **Remark** |
| --- | --- | --- |
| MGG_04503koF1 | TAACTCGAGAGTTGGATCGAGCTGCAGTGTT | amplify *MoCPA2* 5’ flank sequence |
| MGG_04503koF2 | TAAGAATTCGGCTGCAGTATATAGAGTAAAT | amplify *MoCPA2* 5’ flank sequence |
| MGG_04503koF3 | TAAACTAGTATACAGCAATCCTCTTTGAGG | amplify *MoCPA2* 3’ flank sequence |
| MGG_04503koF4 | TAAGAGCTCCATTTCGTCTGCAAGTTCTACC | amplify *MoCPA2* 3’ flank sequence |
| MGG_04503  proconF | GCAATCTGCCCAGCTCGGTGCT | amplify *MoCPA2* probe sequence |
| MGG_04503 proconR | ATGCCGCACGGACGATGATCGGG | amplify *MoCPA2* probe sequence |
| MGG_04503 conF | CAAGAGGAAGGGCATCTTGGAG | validation of *Mocpa2* deletion |
| MGG_04503conR | TCCTCATCAGAGAGGGTCTGAG | validation of *Mocpa2* deletion |
| MGG_04503 BY | CAAGCGTCGATTCGTTTAAAGA | validation of *Mocpa2* deletion (*HPH*) |
| MGG_04503 F | ACTCACTATAGGGCGAATTGGGTACTCAAATTGGTTGCTTTGGAACGTTGGTCGCG | *MoCPA2* complementation |
| MGG_04503R | CACCACCCCGGTGAACAGCTCCTCGCCCTTGCTCACAATAAACTCAGACCACCTGCGG | *MoCPA2* complementation |
| MGG_04503ATPF1 | ACTCACTATAGGGCGAATTGGGTACTCAAATTGGTTGCTTTGGAACGTTGGTCGCG | Construction of *MoCPA2^ΔATP^* |
| MGG_04503ATP F2 | GGATGCCGAGGGGGTCGAAGTTTAGGCGAGGAAGATGCCATCGG | Construction of *MoCPA2^ΔATP^* |
| MGG_04503ATP F3 | CCGATGGCATCTTCCTCGCCTAAACTTCGACCCCCTCGGCATCC | Construction of *MoCPA2^ΔATP^* |
| MGG_04503ATP F4 | CACCACCCCGGTGAACAGCTCCTCGCCCTTGCTCACAATAAACTCAGACCACCTGCGG | Construction of *MoCPA2^ΔATP^* |
| MGG_04503CPS1F1 | ACTCACTATAGGGCGAATTGGGTACTCAAATTGGTTGCTTTGGAACGTTGGTCGCG | Construction of *MoCPA2^ΔCPS1^* |
| MGG_04503CPS1 F2 | ATGTTCTGGAGCTTGTGCAAGACGAGACCAATCTTGGCCGCAGT | Construction of *MoCPA2^ΔCPS1^* |
| MGG_04503CPS1 F3 | ACTGCGGCCAAGATTGGTCTCGTCTTGCACAAGCTCCAGAACAT | Construction of *MoCPA2^ΔCPS1^* |
| MGG_04503CPS1 F4 | CACCACCCCGGTGAACAGCTCCTCGCCCTTGCTCACAATAAACTCAGACCACCTGCGG | Construction of *MoCPA2^ΔCPS1^* |
| MGG_04503CPS2 F1 | ACTCACTATAGGGCGAATTGGGTACTCAAATTGGTTGCTTTGGAACGTTGGTCGCG | Construction of *MoCPA2^ΔCPS2^* |
| MGG_04503CPS2 F2 | GGCAGCACCAGGGTGGCGTCACCAGCGCACCAGTCGAACTCGAC | Construction of *MoCPA2^ΔCPS2^* |
| MGG_04503CPS2 F3 | GTCGAGTTCGACTGGTGCGCTGGTGACGCCACCCTGGTGCTGCC | Construction of *MoCPA2^ΔCPS2^* |
| MGG_04503CPS2 F4 | CACCACCCCGGTGAACAGCTCCTCGCCCTTGCTCACAATAAACTCAGACCACCTGCGG | Construction of *MoCPA2^ΔCPS2^* |
| qRTCPA2F | CTGCTGAGATCGACGTCGAC | quantitative RT-PCR analysis Mo*CPA2* |
| qRTCPA2R | CAGTGATGTTCCAGGCCTTG | quantitative RT-PCR analysis *MoCPA2* |
| Rice_PR1a _QF | TCTTCATCACCTGCAACTACTC | quantitative RT-PCR analysis (PR) genes |
| Rice_PR1a _QR | ATTCATCGGATTTATTCTCACC | quantitative RT-PCR analysis (PR) genes |
| Rice_PBZ1_QF | CTACTATGGCATGCTCAAGAT | quantitative RT-PCR analysis PR genes |
| Rice_PBZ1_QR | ATAGAAAGGCACATAAACACAA | quantitative RT-PCR analysis PR genes |
| Rice_AOS2_QF | CAATACGTGTACTGGTCGAATGG | quantitative RT-PCR analysis PR genes |
| Rice_AOS2_QR | AAGGTGTCGTACCGGAGGAA | quantitative RT-PCR analysis PR genes |
| qRT-actinF | CCATGTACCCTGGTCTTTCG | quantitative RT-PCR analysis ACTIN |
| qRT-actinR | TTCGAGATCCACATCTGCTG | quantitative RT-PCR analysis ACTIN |
